# Comparative Evaluation of AAV8 and AAV9 Gene Therapy in Fabry Knockout (*Gla*^−/y^) and Symptomatic (G3S^Tg/+^*Gla*^−/y^) Murine Models

**DOI:** 10.3390/genes16070766

**Published:** 2025-06-29

**Authors:** Fu-Pang Chang, Ya-Ting Lee, Pao-Hsung Liu, Pei-Sin Chen, Yun-Ru Chen, Dau-Ming Niu

**Affiliations:** 1 Institute of Clinical Medicine, College of Medicine, National Yang Ming Chiao Tung University, Taipei 112304, Taiwan; 2 Department of Pathology and Laboratory Medicine, Taipei Veterans General Hospital, Taipei 11227, Taiwan; 3 Department of Pediatrics, Taipei Veterans General Hospital, Taipei 11217, Taiwan

**Keywords:** Fabry disease, gene therapy, GLA

## Abstract

Background: Fabry disease (FD) is an X-linked lysosomal storage disorder caused by mutations in the GLA gene, resulting in α-galactosidase A (α-Gal A) deficiency and progressive accumulation of globotriaosylceramide (Gb3). Current therapies, such as enzyme replacement and chaperone therapy, have limitations, including incomplete biodistribution and mutation-specific efficacy. Gene therapy using adeno-associated virus (AAV) vectors presents a promising alternative. Methods: In this study, we assessed the dose-dependent effects of AAV8 and AAV9 vectors encoding human GLA in Gla knockout (*Gla*^−/y^) mice by measuring α-Gal A activity and monitoring safety. To evaluate therapeutic efficacy, symptomatic Fabry mice (G3S^Tg/*+*^*Gla*^−/y^) were used. Results: AAV9-GLA produced significantly higher and more sustained enzyme activity than AAV8-GLA across plasma, liver, heart, and kidney. In symptomatic mice, AAV9-GLA achieved superior reductions in serum Gb3 and lyso-Gb3 levels, greater Gb3 clearance in heart and kidney tissues, and improved proteinuria. Anti-GLA IgG titers remained below threshold for the first four weeks and increased modestly by week eight, indicating a limited humoral immune response. No significant clinical signs or weight loss were observed in *Gla*^−/y^ mice over the 3.5-month study period, supporting the favorable safety profile of AAV-mediated gene therapy. Conclusions: These findings demonstrate that AAV9 provides enhanced biodistribution and therapeutic efficacy compared to AAV8, supporting its potential for the treatment of Fabry disease.

## 1. Introduction

Fabry disease (FD; MIM 301500) is an X-linked lysosomal storage disorder caused by mutations in the GLA gene, leading to deficient α-galactosidase A (α-Gal A) activity. This enzyme deficiency results in the progressive accumulation of glycosphingolipids, primarily globotriaosylceramide (Gb3), within lysosomes, particularly in vascular endothelial cells, smooth muscle cells, and renal cells. Lysosomal accumulation of Gb3 leads to cellular dysfunction, contributing to a progressive multisystem disorder affecting the kidneys, heart, and cerebrovascular system. Classic FD presents with early-onset symptoms such as acroparesthesia, angiokeratoma, and hypohidrosis, progressing to severe complications, including renal failure, cardiomyopathy, and stroke in adulthood. More recently, late-onset phenotypes with residual enzyme activity have been recognized, typically manifesting as isolated cardiac or renal involvement in middle-aged or elderly patients [1,2,3,4].

The global incidence of Fabry disease is estimated to range from approximately 1 in 40,000 to 1 in 170,000 [5]; however, Fabry disease carries significant epidemiological relevance in Taiwan. Our research team was the first to identify the high prevalence of a later-onset cardiac variant in Taiwan, associated with the IVS4+919G>A mutation, which affects approximately 1 in 1600 males [6]. This mutation results in aberrant splicing of GLA, leading to markedly reduced α-Gal A enzyme activity and contributing to Fabry cardiomyopathy [7,8,9]. Given this high prevalence, the development of effective therapies that address the cardiac variant of Fabry disease is of particular importance in Taiwan.

Current treatment strategies for Fabry disease include enzyme replacement therapy (ERT) and chaperone therapy. While biweekly intravenous administration of recombinant α-Gal A can slow disease progression, ERT has significant limitations, including a short half-life and suboptimal biodistribution, particularly in cardiac and renal tissues [10,11,12,13]. Chaperone therapy, an oral alternative, is only effective in patients with amenable mutations and requires residual enzyme activity, limiting its applicability [14]. These challenges underscore the need for a more effective and durable therapeutic approach.

Gene therapy using adeno-associated virus (AAV) vectors has emerged as a promising alternative for treating Fabry disease [15,16,17]. AAV vectors provide stable gene expression with a single administration and have demonstrated long-term efficacy in experimental and clinical studies. Among the various AAV serotypes, AAV8 exhibits strong liver tropism [18], enabling high hepatic expression of α-Gal A. The liver’s immune-privileged environment promotes immune tolerance [19], reducing the risk of immune responses compared to enzyme production in muscle or endothelial cells. Additionally, liver-secreted α-Gal A may be taken up by affected cells via the cation-independent mannose 6-phosphate receptor, as seen with ERT [20]. These advantages make AAV8 a suitable choice for this study; however, liver-secreted α-Gal A could potentially share the limitation of suboptimal biodistribution similar to ERT. In contrast, AAV9 has superior heart and kidney tropism and even the ability to cross the blood–brain barrier [21], making it a strong candidate for Fabry gene therapy. Comparative evaluation of these two serotypes can provide valuable insights into optimizing gene therapy strategies for Fabry disease [22,23].

Regarding animal models of Fabry disease, the first Fabry disease mouse model was developed in 1997 by Ohshima et al., who used gene targeting to disrupt the *Gla* gene on the X chromosome, resulting in systemic α-galactosidase A (GLA) deficiency [24]. Although *Gla*^−/y^ mice accumulate globotriaosylceramide (Gb3), similar to Fabry patients, their disease progression is relatively slow, and key clinical features—such as Gb3 accumulation in cardiomyocytes—are absent [25]. To address these limitations, Taguchi et al. generated a more clinically relevant model by crossbreeding human Gb3 synthase (G3S) transgenic mice with Gla knockout mice. The resulting G3S^Tg/+^*Gla*^−/y^ mice exhibit 6–10-fold higher serum Gb3 levels than *Gla*^−/y^ mice and more closely replicate the classic Fabry disease phenotype. They show progressive renal impairment, including early-onset albuminuria starting at 3 weeks of age [26]. This model is therefore preferred for studying pathological manifestations and evaluating therapeutic efficacy. In this study, we used both the *Gla*^−/y^ and G3S^Tg/+^*Gla*^−/y^ models to assess the effects of AAV-mediated gene therapy. The *Gla*^−/y^ mice, which lack endogenous enzyme activity but show no overt clinical symptoms [25], were used to evaluate dose-dependent enzyme expression and to monitor potential safety signals without the confounding influence of disease progression. In contrast, the symptomatic G3S^Tg/+^*Gla*^−/y^ mice, which develop severe manifestations and early mortality [26], were employed to assess therapeutic efficacy by measuring reductions in plasma Gb3 and lyso-Gb3, Gb3 clearance in heart and kidney tissues, and improvements in proteinuria as an indicator of renal function. By comparing outcomes between these two models, we aimed to determine the optimal AAV vector for Fabry gene therapy, with particular emphasis on its potential application in treating the cardiac variant prevalent in Taiwan.

## 2. Materials and Methods

### 2.1. Plasmid Construction and AAV Production

The GLA expression plasmid was constructed using the pXX-UF1-CB-LacZ (B1268) plasmid backbone, which contains a ubiquitous CB promoter (CMV enhancer/chicken β-actin hybrid promoter), a transgene insertion site, and a bovine growth hormone (BGH) polyadenylation signal, flanked by inverted terminal repeats (ITRs). The human GLA transgene was amplified from a previously constructed GLA-myc-6xHis plasmid, preserving the complete 429 amino acid sequence, including the first 31 amino acids that function as a signal peptide for the chimeric protein. The LacZ sequence in the B1268 plasmid was replaced with the GLA transgene, resulting in a recombinant plasmid approximately 6.3 kb in size. Plasmid integrity was confirmed by restriction enzyme digestion using BamHI, SmaI, and NotI. To validate expression and functionality, the B1268-GLA plasmid and an empty plasmid (mock control) were transfected into a GLA-knockout HEK293 cell line. After 48 h, GLA protein expression was confirmed via Western blot using anti-GLA antibodies, and enzyme activity assays verified the functionality of the expressed protein. Approximately 700 μg of the verified plasmid was submitted to the Service of Adeno-associated Viral Vectors, Institute of Biomedical Sciences, Academia Sinica, for AAV8 and AAV9 vector production. Viral genome copy numbers were subsequently quantified by qPCR for use in downstream experiments.

### 2.2. Animals

In the present study, both Fabry disease mouse models on a C57BL/6J background were utilized. *Gla*^−/y^ mice were employed to evaluate AAV vector efficiency, determine optimal dosing, and assess safety, while G3S^Tg/+^*Gla*^−/y^ mice were used to assess therapeutic efficacy. All mice were genotyped by PCR in 7 days after birth and maintained under specific pathogen-free conditions in a controlled environment with a 12 h light/dark cycle and ad libitum access to standard chow and water. All animal procedures were performed in accordance with protocols approved by the Institutional Animal Care and Use Committee (IACUC) of Taipei Veterans General Hospital. For dose-finding and serotype comparison, *Gla*^−/y^ mice were administered recombinant AAV8-GLA or AAV9-GLA viral particles via tail vein injection at doses of 5 × 10^10^, 1 × 10^11^, or 5 × 10^11^ vector genomes (vg) per mouse. Blood, urine, and tissue samples (plasma, liver, heart, brain and kidney) were collected at baseline and designated time points, then stored at −80 °C for subsequent analysis. Based on the dose–response data, a single dose of 1 × 10^11^ vg/mouse was selected for therapeutic evaluation in G3S^Tg/+^*Gla*^−/y^ mice. These symptomatic mice received AAV8-GLA or AAV9-GLA via tail vein injection, and therapeutic efficacy was assessed by measuring GLA enzymatic activity, Gb3 reduction in plasma and target organs, and improvement of proteinuria. Detailed information regarding sample size, mouse age, and group allocation is demonstrated in each figure and summarized in Supplementary Table S1.

### 2.3. Sample Collection

At predetermined endpoints, mice were euthanized using carbon dioxide (CO_2_) inhalation, and death was confirmed by applying a nociceptive stimulus. Cardiac blood was collected via transcardial puncture using a 26G 1 mL syringe inserted through the xiphoid process. Following needle removal, blood was transferred into EDTA-coated tubes and gently mixed. Plasma was separated by centrifugation at 13,200 rpm for 10 min at 4 °C, and the supernatant was aliquoted into 1.5 mL microcentrifuge tubes and stored at −80 °C until analysis. Subsequently, tissue samples were harvested. The abdominal cavity was opened using sterilized scissors and forceps disinfected with 70% ethanol. Target organs were excised, trimmed to appropriate sizes, and washed briefly in phosphate-buffered saline (PBS) to remove residual blood. Excess moisture was blotted using sterile tissue paper, and samples were transferred into 2 mL microcentrifuge tubes, aliquoted as necessary, and stored at −80 °C. For urine collection, mice were individually placed on a sterile 96-well plate positioned within a clean plastic chamber. Following confirmation of toe markings for identification, mice were allowed to void naturally. Urine was collected using sterile pipette tips and transferred to 1.5 mL microcentrifuge tubes. Samples were stored at −80 °C for up to two months prior to downstream analysis. The detailed study design is described in Supplementary Figure S1.

### 2.4. Activity Assay of α-Gal A

Fluorometric assay of α-Gal A was performed as described, with minor modifications [27]. Briefly, tissue samples were homogenized and sonicated in 0.1% Triton X buffer at a ratio of 0.1 g/mL and then centrifuged at 13,200 rpm for 10 min. α-Gal A activity was determined by incubating aliquots of the supernatant solution at 37 °C with 5 mM 4-methylumbelliferyl-α-D-galactopyranoside in C-P buffer (28 mM citric acid, 44 mM disodium phosphate, 5 mg/mL sodium taurocholate, pH 4.4) and the presence of 0.1 M N-acetylgalactosamine, a specific inhibitor of N-acetylgalactosaminidase. Protein concentrations were determined by the method of Bio-Rad Protein Assay (BioRad #500-0006). One unit of α-Gal A activity is equivalent to the hydrolysis of 1 nmol of substrate in 1 h at 37 °C.

### 2.5. Quantitation of Gb3 Levels

Blood samples were collected from mice by puncturing the facial vein using a lancet. The blood was allowed to drip into 1.5 mL microcentrifuge tubes and left at room temperature for approximately 10 min to allow clotting. The samples were then centrifuged at 4600 rpm for 10 min at 4 °C, and the resulting serum was carefully transferred to new microcentrifuge tubes, aliquoted appropriately, and stored at –80 °C until further analysis. Gb3 and lyso-Gb3 levels in serum were quantified via tandem mass spectrometry. Briefly, 50 μL of serum samples, calibration standards, and internal standards were added to a 96-well plate, followed by lipid extraction using a mixture of chloroform, methanol, and formic acid with multiple centrifugation steps. Stepwise gradient elution was performed using a Waters Alliance 2795XE HPLC system (USA), with detection carried out on a triple quadrupole mass spectrometer (Quattro Ultima, Waters, Milford, MA, USA) operating in multiple reaction monitoring (MRM) mode. Data were processed using NeoLynx software version 4.1. Quality control was maintained using lyso-Gb3–spiked plasma at 5 nM (low) and 100 nM (high) concentrations, with inter-assay coefficients of variation (CV) of 9.2% for the high control and 11.0% for the low control, and intra-assay CVs of 12.8% and 9.9%, respectively. The analytical methodology remained consistent with our previously published protocol for human samples [28].

### 2.6. Immunofluorescent Staining of Gb3

Immunofluorescent staining of Gb3 was performed according to the method described, with some modifications [29]. Frozen-sectioned tissues were fixed with Cytofix/Cytoperm™ (BD #554722) for 20 min. After removal of fixing solution, the specimens were rinsed with Perm/wash buffer (BD #554723) and blocked with 5% FBS Perm/wash buffer overnight at 4 °C. The specimens were then incubated with Anti-human CD77 Antibody (Mouse IgM, BioLegend #357101) at 4 °C overnight, followed by washing with Perm/wash buffer. Specific binding was visualized with FITC Anti-mouse IgM antibody (BioLegend #406505). Slides were also stained with DAPI (Sigma # D9542) before being analyzed under a fluorescent microscope. Image quantification was performed using ImageJ software (Version 1.54p, National Institutes of Health, USA). Graphs and statistical analyses were generated using GraphPad Prism software. Details of the statistical methods are provided in Section 2.9.

### 2.7. Analysis of Proteinuria

Proteinuria was determined by quantitation of the urine microalbumin to creatinine ratio. Murine microalbuminuria and creatinine were determined using A murine microalbuminuria ELISA Kit (Exocell #1011) and Creatinine companion Kit (Exocell #1012), according manufacturer’s protocol.

### 2.8. Anti-α-Gal A Immune Response

Sera were collected at designated time points, and antibody responses against a-gal A were measured by ELISA as described with some modifications [30]. Briefly, 96-well, flat-bottom microtiter plates were coated with α-Gal A (4 μg/mL) in coating buffer (0.05 M Na_2_CO_3_, pH 9.5) and incubated overnight at 4 °C. After washing in 0.05% Tween-PBS, plates were blocked in 2.5%BSA- PBS-sodium azide buffer overnight at 4 °C. Subsequently, sera were serially diluted in antibody buffer (0.5% BSA-PBS, pH 8.0) and incubated for 2 h at 37 °C. Different concentrations of mouse anti-human GLA IgG (Proteintech #66121-1-Ig) were used to form a standard curve. After washing, alkaline phosphatase-conjugated goat anti-mouse IgG (GeneTex #21311101) was added in a volume of 100 μL (500 ng/mL) per well and incubated for 1 h at 37 °C. TMB substrate (BioGenex #QD420-YIKE) was added (100 μL/well) and incubated for 10 min. 1N HCL was then added as a stop solution. The absorbance at 450 nm was determined with a SpectraMax M5 multi-detection reader (Molecular Devices, San Jose, CA, USA), and the amount of antibody was calculated according to the standard curve.

### 2.9. Statistical Analysis

All statistical analyses were performed using GraphPad Prism software (version 5.01). Non-parametric tests were used to account for potential deviations from normality and unequal variances. For comparisons involving more than two groups, the Kruskal–Wallis test followed by Dunn’s post hoc test was applied. For comparisons between two groups, the Mann–Whitney U test (also known as the Wilcoxon rank-sum test) was used. A *p*-value of less than 0.05 was considered statistically significant. Asterisks in figures indicate the following levels of significance: *p* < 0.05 (*), *p* < 0.01 (**), and *p* < 0.001 (***). Data are presented as mean ± standard deviation (SD), and the number of biological replicates (n) is indicated in each figure legend. Animals were randomly assigned to treatment groups. Three researchers were involved in the experimental procedures, each responsible for different technical aspects, including vector administration, tissue collection, and biochemical analysis. To ensure blinding, individual investigators were not aware of the group assignments handled by others.

## 3. Results

### 3.1. Dose-Dependent GLA Enzyme Activity Following AAV8-GLA and AAV9-GLA Gene Therapy in Fabry Mice

**Figure 1:**
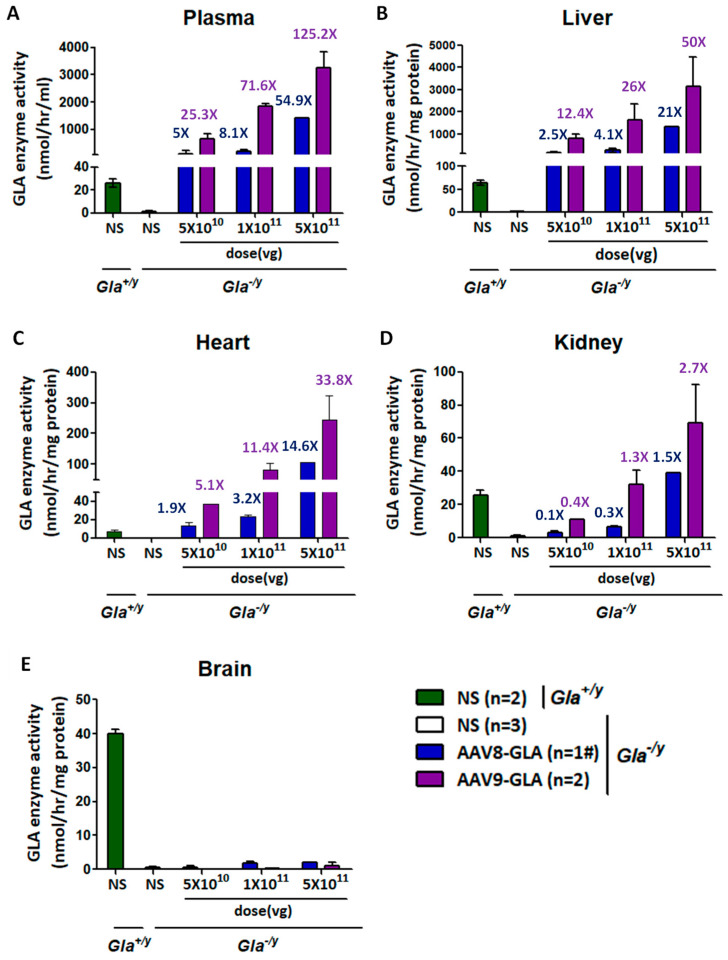
Dose-dependent GLA enzymatic activity in circulation and tissues of *Gla*^−/y^ mice following AAV8-GLA and AAV9-GLA single-dose administration gene therapy. WT(*Gla*^+/y^) and *Gla*^−/y^ male mice (4-6 months old) received a single intravenous injection of AAV8-GLA or AAV9-GLA at low, medium, or high doses (5 × 10^10^, 1 × 10^11^, 5 × 10^11^ vg/mouse) for 3 weeks. GLA enzymatic activity was measured three weeks post-injection in (**A**) plasma, (**B**) liver, (**C**) heart, (**D**) kidney, and (**E**) brain. Data are presented as mean ± SD. #: In this group, two out of three treated mice died shortly after injection; therefore, data from only one mouse was available, and standard deviation could not be calculated.

Based on prior studies demonstrating that AAV-mediated transgene expression reaches a steady state within 2–4 weeks post-injection in murine models [31,32,33], a three-week dose-finding study was conducted in 4- to 6-month-old male *Gla*^−/y^ Fabry mice. Single tail vein injections of recombinant AAV8-GLA or AAV9-GLA vectors at doses of 5 × 10^10^, 1 × 10^11^, or 5 × 10^11^ vg/mouse resulted in dose-dependent increases in GLA enzyme activity across plasma, liver, heart, and kidney (Figure 1). Compared to wild-type controls, significantly elevated enzyme activity was observed even at the lowest dose for both vectors, with AAV9-GLA consistently inducing higher activity than AAV8-GLA. At the highest AAV9-GLA dose, enzyme activity increased approximately 125.2-fold in plasma (3242.68 ± 600.32 vs. 25.9 ± 3.68 nmol/h/mL), 50-fold in liver (3155.31 ± 1325.35 vs. 63.17 ± 5.59 nmol/h/mg protein), and 33.8-fold in heart (245.18 ± 78.12 vs. 7.25 ± 1.34 nmol/h/mg protein) relative to wild-type mice. In the kidney, only AAV9-GLA-treated mice in the medium- and high-dose groups, and AAV8-GLA-treated mice in the high-dose group, achieved enzyme activity levels comparable to wild-type controls (Figure 1D). Notably, in the high-dose AAV8-GLA group (5 × 10^11^ vg/mouse), two out of three treated *Gl*a^−/y^ mice died shortly after injection, raising safety concerns at this dose level. Given that the intermediate dose (1 × 10^11^ vg/mouse) already achieved robust and therapeutically relevant enzyme activity across target tissues, this dose was selected for all subsequent in vivo experiments to balance efficacy and safety.

Regarding gene transduction efficiency, quantification of viral genome copy number revealed that both AAV8-GLA and AAV9-GLA vectors achieved relatively high copy numbers in the liver. Moreover, AAV9-GLA consistently exhibited higher genome copy numbers than AAV8-GLA, particularly at the highest dose (Supplemental Figure S2). However, neither viral genome copy number nor enzyme activity was detected in brain tissue across any of the treatment groups (Figure 1E).

### 3.2. Sustained Enzyme Activity Three Months Post-Treatment

**Figure 2:**
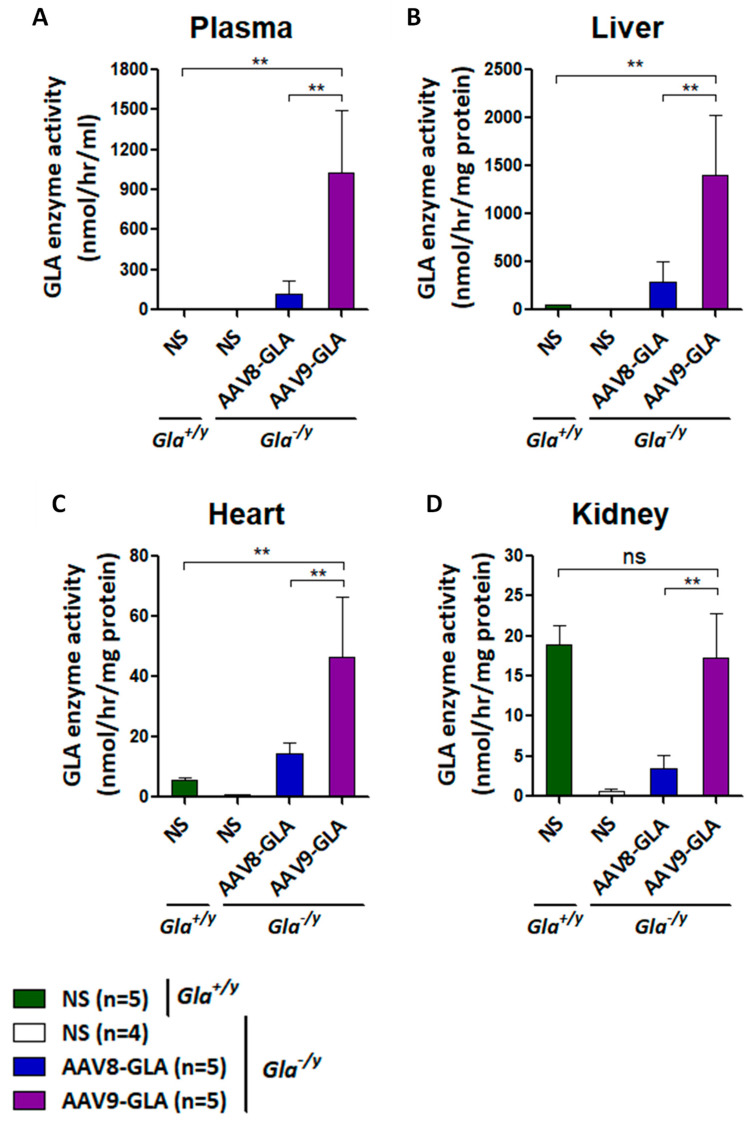
Sustained GLA enzymatic activity in *Gla*^−/y^ mice three months after AAV8-GLA or AAV9-GLA administration. WT(*Gla*^+/y^) and *Gla*^−/y^ male mice (2–4 months old) were treated with a single intravenous injection of AAV8-GLA or AAV9-GLA at 1 × 10^11^ vg/mouse. GLA activity was measured three months post-injection in (**A**) plasma, (**B**) liver, (**C**) heart, and (**D**) kidney. Data are pre-sented as mean ± SD. *p* < 0.01 (**).

To evaluate long-term expression, a medium dose (1 × 10^11^ vg/mouse) of AAV8-GLA or AAV9-GLA was administered to 2–4-month-old *Gla*^−/y^ mice. Analysis of enzyme activity in plasma, liver, heart, and kidney at three months post-treatment revealed sustained expression across all tissues for both AAV8-GLA and AAV9-GLA vectors (Figure 2). Notably, AAV9-GLA consistently maintained significantly higher enzyme activity than AAV8-GLA, with levels measured in plasma (1030.30 ± 465.22 vs. 122.84 ± 98.33 nmol/h/mL), liver (1405.22 ± 616.18 vs. 284.85 ± 217.08 nmol/h/mg protein), heart (46.30 ± 20.26 vs. 14.34 ± 3.66 nmol/h/mg protein), and kidney (17.31 ± 5.57 vs. 3.54 ± 1.54 nmol/h/mg protein, *p* < 0.005). Kidney enzyme activity in AAV9-GLA-treated mice remained at levels comparable to wild-type controls (18.91 ± 2.34 nmol/h/mg protein). These findings demonstrate that AAV9-GLA provides superior and sustained enzyme expression in multiple organs.

### 3.3. GLA Enzyme Expression in Symptomatic Fabry Mice Treated with AAV8-GLA or AAV9-GLA

**Figure 3:**
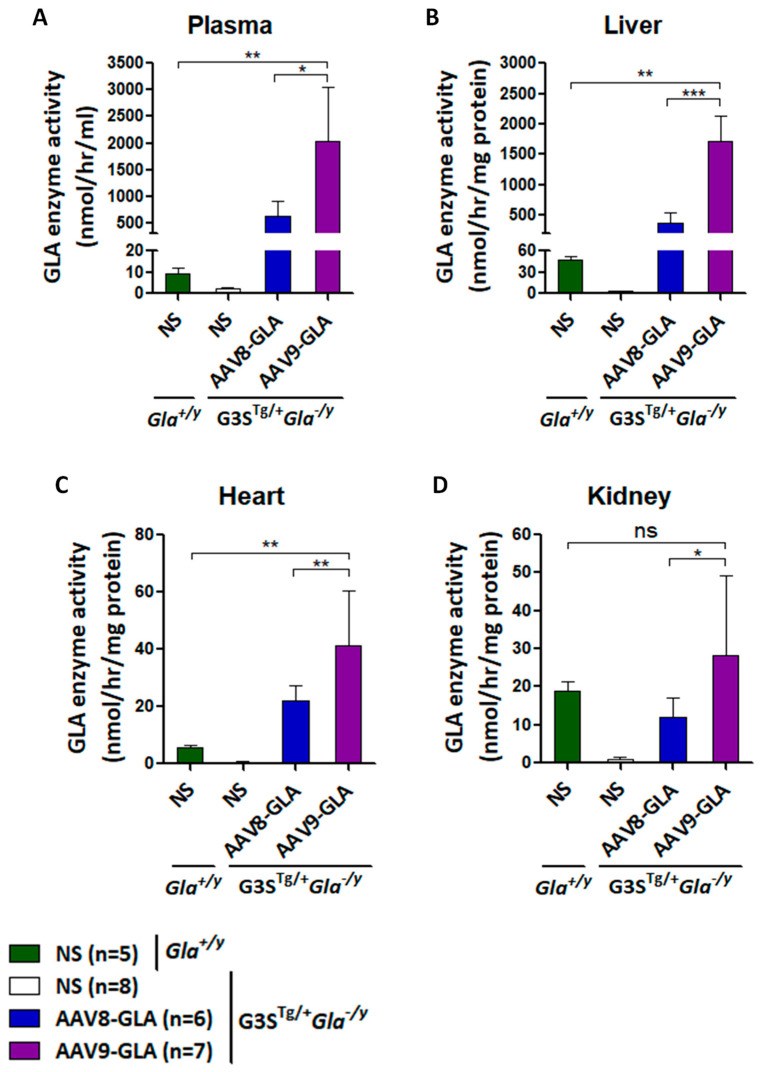
GLA enzymatic activity in G3S^Tg/+^*Gla*^−/y^ mice following 3.5 months of AAV8-GLA or AAV9-GLA treatment. WT and G3S^Tg/+^*Gla*^−/y^ male mice (2–3 months old) received a single intravenous injection of AAV8-GLA or AAV9-GLA at 1 × 10^11^ vg/mouse. GLA activity was assessed at 3.5 months post-injection in (**A**) plasma, (**B**) liver, (**C**) heart, and (**D**) kidney. Data are presented as mean ± SD. *p* < 0.05 (*), *p* < 0.01 (**), *p* < 0.001 (***).

To evaluate the therapeutic efficacy of AAV-GLA gene therapy in a symptomatic model, we used G3S^Tg/+^*Gla*^−/y^ mice, which closely mimic classical Fabry disease through elevated serum Gb3, organ accumulation, and progressive renal impairment. Mice aged 2–3 months were randomly assigned to receive a medium dose (1 × 10^11^ vg/mouse) of AAV8-GLA or AAV9-GLA, with untreated mice serving as controls. At three months post-injection, both vectors maintained stable enzyme activity in plasma, liver, heart, and kidney; however, AAV9-GLA consistently achieved significantly higher activity compared to AAV8-GLA and baseline *Gla*^+/y^ (wild type) mice (*p* < 0.005) (Figure 3). In the heart, AAV9-GLA-treated mice reached enzyme levels of 41.36 ± 19.26 nmol/h/mg, significantly exceeding those of AAV8-GLA (22.07 ± 5.20 nmol/h/mg) and *Gla*^+/y^ controls (5.57 ± 0.86 nmol/h/mg). Notably, AAV9-GLA also produced higher enzyme activity in kidney tissue (28.28± 21.00 nmol/h/mg) compared to AAV8-GLA (11.91 ± 5.09 nmol/h/mg, *p* < 0.05), overcoming prior challenges in kidney transduction (Figure 3D).

### 3.4. Reduction In Serum Gb3 and Lyso-Gb3 in Symptomatic Fabry Mice Following AAV8-GLA or AAV9-GLA Treatment

**Figure 4:**
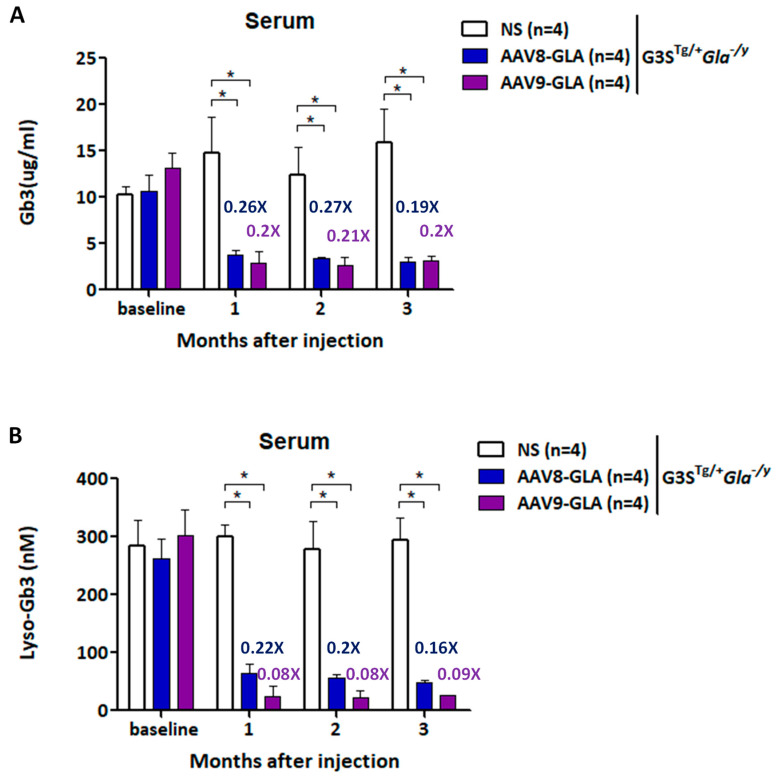
Reduction in plasma Gb3 and lyso-Gb3 levels in G3S^Tg/+^*Gla*^−/y^ mice following AAV-mediated gene therapy. WT and G3S^Tg/+^*Gla*^−/y^ male mice (2-3 months old) were treated with a single intravenous dose of AAV8-GLA or AAV9-GLA at 1 × 10^11^ vg/mouse. Levels of (**A**) Gb3 and (**B**) lyso-Gb3 in plasma were analyzed 3.5 months post-injection. Data are presented as mean ± SD. *p* < 0.05 (*).

To assess Gb3 clearance efficacy, we measured serum Gb3 and lyso-Gb3 levels at 1, 2, and 3 months post-treatment (Figure 4). As early as one month after treatment, both AAV8-GLA and AAV9-GLA groups demonstrated significantly reduced serum Gb3 to approximately 20% of untreated levels (*p* < 0.05). More impressively, serum lyso-Gb3 levels decreased to 22% for AAV8-GLA and 8% for AAV9-GLA compared to untreated controls (*p* < 0.05), with AAV9-GLA showing significantly greater clearance than AAV8-GLA (*p* < 0.05) (Figure 4B). Importantly, the reductions in both Gb3 and lyso-Gb3 were sustained throughout the entire 3-month study period.

### 3.5. Comparison of Gb3 Clearance in Renal and Cardiac Tissues of Symptomatic Fabry Mice Treated with AAV8-GLA or AAV9-GLA

**Figure 5:**
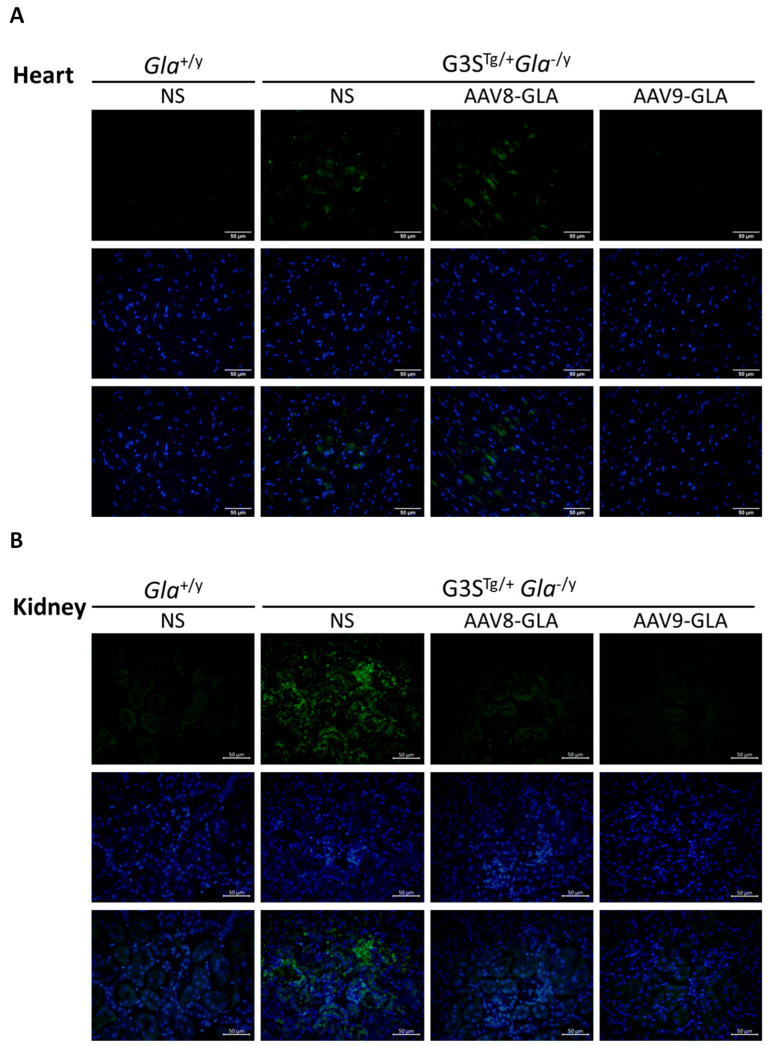

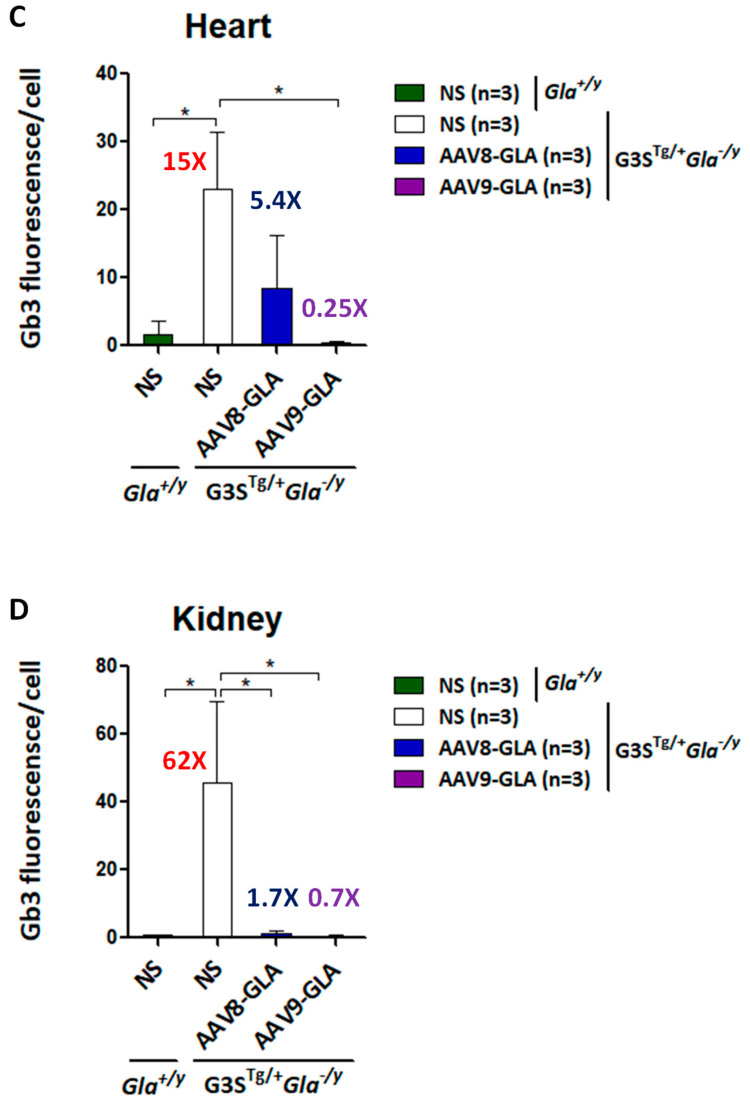
Clearance of Gb3 from heart and kidney tissues in G3S^Tg/+^*Gla*^−/y^ mice following 3.5 months of AAV-GLA treatment. WT and G3S^Tg/+^*Gla*^−/y^ male mice (2–3 months old) received a single intravenous injection of AAV8-GLA or AAV9-GLA at 1 × 10^11^ vg/mouse. Gb3 clearance was evaluated in tissue sections of (**A**) heart and (**B**) kidney by immunofluorescence using anti-Gb3 antibody and DAPI. Quantification of (**C**) cardiac and (**D**) renal Gb3 fluorescence intensity per cell was performed using ImageJ. Data are presented as mean ± SD. *p* < 0.05 (*).

To evaluate the efficacy of gene therapy in clearing Gb3 accumulation, we performed immunofluorescence staining and quantitative analysis of heart and kidney tissues at 3.5 months post-injection. Untreated G3S^Tg/+^*Gla*^−/y^ mice demonstrated significantly higher Gb3 accumulation in both heart and kidney tissues compared to wild-type *Gla*^+/y^ mice (*p* <0.05) (Figure 5, Supplentary Figure S5). Both AAV8-GLA and AAV9-GLA treatments showed significant Gb3 clearance compared to untreated mice, with AAV9-GLA exhibiting superior therapeutic effects. In the heart, AAV9-GLA treatment not only significantly reduced Gb3 accumulation (*p* <0.05) but also restored Gb3 levels to those observed in normal mice, outperforming AAV8-GLA. Similarly, in kidney tissues, both AAV8-GLA and AAV9-GLA treatments resulted in substantial Gb3 clearance (*p* <0.05), effectively returning Gb3 levels to those of wild-type mice. These findings demonstrate that while untreated mice exhibit progressive Gb3 accumulation with age, AAV-mediated gene therapy, particularly AAV9-GLA, can effectively mitigate Gb3 accumulation in critical organ systems, with a more pronounced effect in cardiac tissues.

### 3.6. Comparison of Proteinuria Response in Symptomatic Fabry Mice Treated with AAV8-GLA or AAV9-GLA

**Figure 6:**
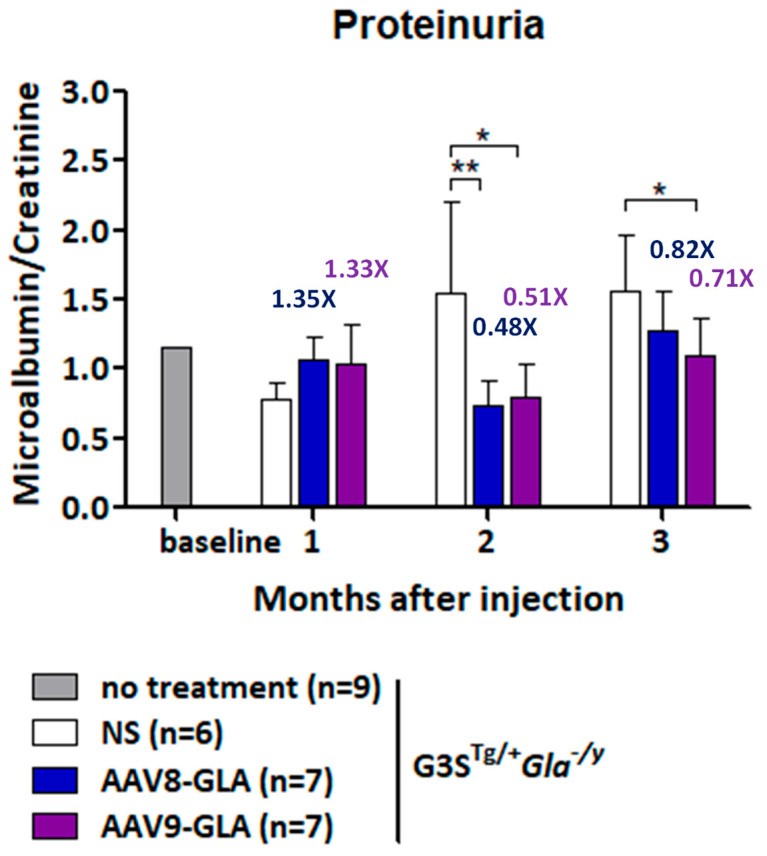
Longitudinal analysis of urinary protein levels in G3S^Tg/+^*Gla*^−/y^ mice after AAV-GLA gene therapy. G3S^Tg/+^*Gla*^−/y^ male mice (2-3 months old) were treated with AAV8-GLA or AAV9-GLA at 1 × 10^11^ vg/mouse. Urine samples were collected before treatment and monthly for three months post-treatment to determine the albumin-to-creatinine ratio. Data are presented as mean ± SD. *p* < 0.05 (*), *p* < 0.01 (**).

To assess the impact of gene therapy on renal function, we analyzed proteinuria progression by measuring the urinary albumin-to-creatinine ratio (ACR) at 1, 2, and 3 months post-treatment with a medium dose (1 × 10^11^ vg/mouse) of AAV-GLA vectors (Figure 6). Untreated G3S^Tg/+^*Gla*^−/y^ mice exhibited a progressive increase in proteinuria, with ACR rising from 0.78 ± 0.11 at 8–12 weeks to 1.56 ± 0.41 at 20–24 weeks, representing a 2-fold elevation. During the first month of treatment, no significant differences in ACR were observed between groups. However, by the second month, both AAV8-GLA and AAV9-GLA treatments significantly reduced ACR by 1.95- to 2.1-fold compared to untreated controls (*p* < 0.01), with values decreasing from 1.54 ± 0.67 in untreated mice to 0.74 ± 0.17 in the AAV8-GLA group and 0.79 ± 0.24 in the AAV9-GLA group. At three months, AAV-GLA treatment continued to suppress proteinuria, with ACR reduced by 1.22- to 1.41-fold compared to untreated controls (untreated: 1.56 ± 0.41; AAV8-GLA: 1.28 ± 0.28; AAV9-GLA: 1.10 ± 0.26). Notably, AAV9-GLA treatment achieved a statistically significant reduction in proteinuria at this time point (*p* < 0.05). These findings demonstrate that AAV-mediated gene therapy, particularly AAV9-GLA, can mitigate the progression of proteinuria, a key marker of renal dysfunction in Fabry disease.

### 3.7. AAV-Mediated Gene Therapy Exhibits Limited Immunogenicity and a Favorable Safety Profile in Fabry Disease Mice

**Figure 7:**
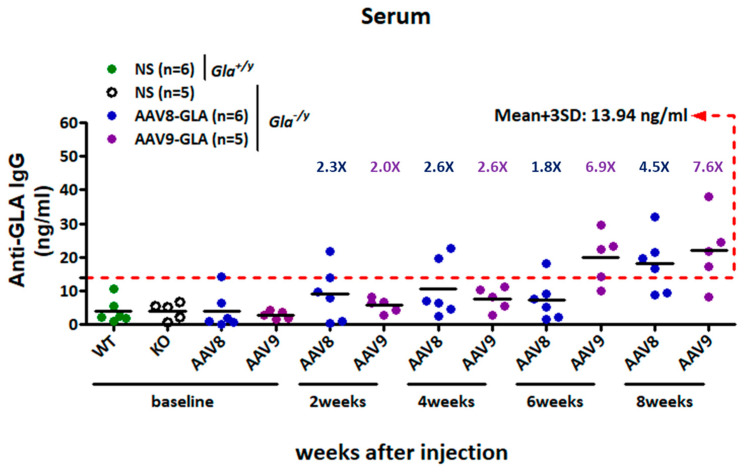
Development of anti-GLA IgG responses in *Gla*^−/y^ mice following AAV8-GLA or AAV9-GLA administration. Gla^−/y^ male mice (2–4 months old) received a single intravenous injection of AAV8-GLA or AAV9-GLA at 1 × 10^11^ vg/mouse. Serum anti-GLA IgG titers were measured at baseline and at weeks 2, 4, 6, and 8 post-injection. Sera from untreated *Gla*^−/y^ mice were used to define the assay cutoff, calculated as the mean plus three standard deviations (mean + 3 SD), and indicated by a red dashed line.

To evaluate the immunogenic and safety profile of AAV-mediated gene therapy, *Gla*^−/y^ mice received a single medium dose (1 × 10^11^ vg/mouse) of AAV8-GLA or AAV9-GLA via tail vein injection. Saline-injected *Gla*^−/y^ and wild-type *Gla*^+/y^ mice served as controls. Serum anti-GLA IgG antibody titers were measured at baseline and at 2, 4, 6, and 8 weeks post-injection. Using a predefined cut-off value (mean + 3 SD = 13.94 ng/mL) based on untreated *Gla*^−/y^ sera, both treatment groups maintained titers below the threshold during the first four weeks (AAV8-GLA: 10.57 ± 8.48 ng/mL; AAV9-GLA: 7.63 ± 3.54 ng/mL). By week 8, titers modestly exceeded the cut-off (AAV8-GLA: 18.14 ± 8.62 ng/mL; AAV9-GLA: 22.00 ± 10.88 ng/mL), suggesting a limited humoral immune response to the transgene (Figure 7).

To assess safety, body weight was monitored weekly for 3.5 months after treatment. Both AAV8-GLA- and AAV9-GLA-treated *Gla*^−/y^ mice maintained stable body weights throughout the study. By week 14 post-injection (corresponding to 22–26 weeks of age), treated mice showed no significant clinical signs or weight loss and remained comparable to untreated *Gla*^−/y^ and wild-type *Gla*^+/y^ controls, supporting the favorable safety profile of this gene therapy approach (Supplemental Figure S3A).

Despite robust expression of GLA enzyme activity and effective clearance of Gb3 in key peripheral organs—including the heart, liver, and kidney—AAV treatment did not significantly extend the lifespan of G3S^Tg/+^*Gla*^−/y^ mice compared with their untreated littermates. In a long-term follow-up cohort, animals began to exhibit significant weight loss around 4–5 months post-injection, ultimately exceeding the 20% threshold set by IACUC guidelines and necessitating euthanasia (Supplementary Figure S3B). These weight declines were observed in both untreated and AAV9-treated G3S^Tg/+^*Gla*^−/y^ mice and are reflected in the corresponding survival curve (Supplementary Figure S4). Although no statistically significant survival advantage was observed in the treated group, one AAV9-treated mouse remained alive through the study’s 9-month endpoint, while all G3S^Tg/+^*Gla*^−/y^ mice had succumbed before 6 months of age. In contrast, wild-type and *Gla*^−/y^ mice—regardless of treatment—exhibited stable body weights and 100% survival, further supporting the safety of the administered AAV doses. The limited therapeutic benefit observed in G3S^Tg/+^*Gla*^−/y^ mice is likely attributable to central nervous system (CNS) involvement, which was not ameliorated by systemic AAV administration.

## 4. Discussion

In this study, we conducted a comparative evaluation of two adeno-associated virus (AAV) serotypes, AAV8 and AAV9, for gene therapy in Fabry disease using both *Gla*^−/y^ knockout and G3S^Tg/+^*Gla*^−/y^ symptomatic murine models. Our results demonstrate that AAV9-GLA provides superior transduction efficiency, enzyme activity, substrate clearance, and therapeutic efficacy compared to AAV8-GLA, while maintaining a favorable safety and immunogenicity profile.

We first assessed the dose-dependent effects of AAV8-GLA and AAV9-GLA in *Gla*^−/y^ mice. In both vectors, α-Gal A expression levels increased proportionally with the administered dose, demonstrating clear dose-dependency. Although AAV8 primarily targets the liver, at equivalent vector doses, AAV9 still induced higher and more sustained levels of α-Gal A activity in the liver. Furthermore, AAV9 achieved higher α-Gal A expression in plasma and across major organs, including the heart and kidney. This finding is consistent with prior reports of AAV9’s enhanced tissue tropism, particularly for cardiac and renal tissues, which are critical therapeutic targets in Fabry disease.

In the symptomatic G3S^Tg/+^*Gla^−^*^/y^ model, which recapitulates key clinical features of classical Fabry disease, AAV9-GLA again outperformed AAV8-GLA. AAV9 treatment led to significantly greater reductions in serum Gb3 and lyso-Gb3 levels, more effective clearance of Gb3 in heart and kidney tissues, and improved renal function, as reflected by decreased proteinuria. These results confirm the functional advantage of AAV9-mediated transduction in disease-relevant tissues and provide strong support for its use in therapeutic applications.

In our observations, even three months after injection, the AAV8-GLA group exhibited approximately fourfold higher α-Gal A activity in the heart compared to wild-type mice. However, despite this elevated enzyme activity, AAV8 treatment did not effectively clear Gb3 accumulation in cardiac tissue, unlike AAV9 treatment, which achieved near-complete Gb3 clearance in myocardial biopsies. These findings suggest that while AAV8 primarily targets the liver and provides systemic distribution of secreted enzyme, liver-derived α-Gal A may be insufficient for optimal cardiac correction. In contrast, AAV9, with its intrinsic tropism for heart tissues, achieved better local expression and more effective clinical outcomes, highlighting the superior potential of AAV9-mediated gene therapy compared to liver-derived enzyme or even current enzyme replacement therapy (ERT) for addressing Fabry disease pathology.

AAV9 is well known to be capable of crossing the blood–brain barrier (BBB) following intravenous (IV) injection, as demonstrated in neonatal mice and human infants [21,34,35]. Maria Grazia Biferi et al. showed that AAV9-GLA could cross the BBB in a Fabry *Gla*^−/y^ mouse model [36]. However, in our current study, AAV9 exhibited very poor BBB penetration in symptomatic G3S^Tg/+^*Gla*^−/y^ Fabry mice, consistent with the findings of Yuka Hayashi et al. [22,23]. The reasons for the limited BBB crossing observed in symptomatic G3S^Tg/+^*Gla*^−/y^ mice remain unclear. Notably, when comparing these three studies, Biferi et al. utilized a much higher vector dose (3.2 × 10^14^ vg/kg) compared to the doses used in our study (5 × 10^11^ vg/mouse) and in Hayashi’s study (2 × 10^12^ vg/mouse), suggesting that vector dose may play a critical role in enabling BBB transduction. In addition, age-related differences in blood–brain barrier (BBB) maturation may also contribute to this variability. AAV9 is known to cross the BBB more efficiently in neonatal or very young mice [34], whereas BBB permeability is markedly reduced in juvenile and adult mice. Therefore, further investigation is warranted to clarify the age-dependent effects and explore whether early-stage AAV9 gene therapy could enhance CNS delivery or whether additional, as yet unidentified, mechanisms are involved.

One major limitation of this study is the presence of species-specific differences between the G3S^Tg/+^*Gla*^−/y^ mouse model and human Fabry patients. Although the G3S^Tg/+^*Gla*^−/y^ model successfully reproduces several key pathological features of Fabry disease—including lysosomal Gb3 accumulation, functional renal impairment, and the development of neurological symptoms—important differences remain, particularly in the distribution of Gb3 and the resulting clinical manifestations in the central nervous system (CNS) and kidneys. In human Fabry disease, CNS involvement is primarily vascular in origin. Clinically and radiologically, patients often present with dolichoectasia of the vertebrobasilar arteries, the pulvinar sign on T1-weighted MRI, as well as white matter hyperintensities, lacunar infarcts, and cerebral microbleeds. These manifestations are widely believed to result from Gb3 accumulation in cerebral endothelial cells, leading to microangiopathy, impaired cerebral perfusion, and increased stroke risk. While some neuronal Gb3 storage may be observed, it is typically not prominent, and overt neurological symptoms—such as tremors, gait disturbances, or kyphosis—are rare in Fabry patients. In contrast, the G3S^Tg/+^*Gla*^−/y^ mouse exhibits extensive Gb3 accumulation in brain parenchymal cells, particularly neurons and glial cells, as confirmed by immunohistochemical staining [24]. This is accompanied by early-onset and severe neurological abnormalities, including spontaneous tremors, kyphosis, and abnormal gait, which typically begin around 20 weeks of age. These prominent neurobehavioral phenotypes, which are uncommon in human Fabry disease, suggest that CNS abnormalities in the mouse model may involve direct neuronal injury and therefore may not accurately reflect the vascular-centric CNS pathology observed in human patients.

A similar divergence is observed in renal pathology. In human Fabry disease, Gb3 primarily accumulates in glomerular podocytes, endothelial cells, and mesangial cells, leading to proteinuria, progressive glomerulosclerosis, and decline in glomerular filtration rate. In contrast, the G3S^Tg/+^*Gla*^−/y^ mouse shows a distinct distribution pattern, with some Gb3 accumulation in glomeruli but predominantly in renal tubular epithelial cells [24]. This leads to impaired urinary concentrating ability, increased urine volume, and reduced urine osmolality. Although this model recapitulates certain functional impairments, its predominant involvement of renal tubules—rather than glomeruli—limits its ability to fully mirror the renal manifestations of human Fabry nephropathy.

Due to current limitations in funding and manpower, we prioritized the assessment of proteinuria—an important marker of renal involvement in human Fabry disease—as the primary focus of this study. Additional disease-relevant parameters, such as urine osmolality and detailed kidney histology, were not evaluated in this work, although they have been included in the original characterization of the Fabry mouse model by Taguchi et al. [24]. Another important limitation of this study is the absence of functional cardiac assessments, despite the heart being a major target organ in Fabry disease. Due to equipment constraints and the limited blood volume that can be safely obtained from small animals, echocardiographic measurements and cardiac biomarker analyses (e.g., serum troponin) were not performed. Nevertheless, previous studies have documented several early cardiomyopathic features in *Gla*^−/y^ (*Gla*KO) mice, including mild cardiomegaly characterized by up to a 25% increase in left ventricular (LV) mass, without significant LV wall thickening [37]. Notably, a significant widening of the LV internal diameter—up to a 24% increase—was observed in 9-month-old *Gla*^−/y^ mice compared to age-matched wild-type controls [37]. These findings highlight the importance of incorporating cardiac imaging and functional assessments in future studies to comprehensively evaluate the therapeutic effects of AAV-mediated gene therapy on Fabry-related cardiomyopathy. Nevertheless, our findings clearly demonstrate a robust increase in α-Gal A activity across multiple tissues and a marked reduction in Gb3 accumulation, supporting the therapeutic potential of this AAV-mediated gene therapy approach. In future studies, we aim to incorporate comprehensive evaluations of both cardiac and renal function to more fully assess therapeutic efficacy in this Fabry disease model.

Immunogenicity remains a major concern in gene therapy. In our study, anti-GLA IgG levels stayed below the defined threshold during the first four weeks and increased only modestly by week eight, suggesting a limited humoral immune response. Although neutralizing antibody activity was not directly assessed, the absence of significant clinical signs or weight loss in treated *Gla*^−/y^ mice throughout the 9-month study period supports the overall safety of both AAV vectors. These findings are encouraging for the future clinical translation of Fabry gene therapy.

In conclusion, our results demonstrate that AAV9-GLA gene therapy achieves superior tissue biodistribution, enzymatic activity, and therapeutic efficacy compared to AAV8-GLA in both *Gla*^−/y^ and G3S^Tg/+^*Gla*^−/y^ mouse models of Fabry disease. Although the major CNS manifestations in human Fabry disease are believed to result primarily from vascular endothelial involvement, Gb3 inclusions have also been identified in neurons upon histopathological examination [23]. Therefore, continued development of AAV9-based gene therapy strategies is warranted—particularly with efforts to enhance CNS delivery through capsid engineering or alternative routes of administration. Future directions will focus on optimizing vector dose and timing, improving tissue-specific transduction, implementing immune modulation strategies, prolonging therapeutic durability, and expanding CNS and cardiac targeting. Collectively, these advances will facilitate clinical translation and support the development of a more comprehensive and effective gene therapy for Fabry disease.

## Data Availability

The original contributions presented in this study are included in the article/Supplementary Materials. Further inquiries can be directed to the corresponding author.

## Supplementary Materials

The following supporting information can be downloaded at: https://www.mdpi.com/article/10.3390/genes16070766/s1, Figure S1: Experimental design overview; Figure S2: Viral vector bio-distribution in tissues of *Gla*^−/y^ mice following AAV-GLA administration; Figure S3: Body weight monitoring of G3S^Tg/+^*Gla*^−/y^ mice during 3.5 months (A) and 5 months (B) of AAV-GLA treatment; Figure S4: Survival curve of mice monitored over 9 months of AAV9-GLA treatment; Figure S5: Additional figure demonstrating Gb3 Clearance in Heart and Kidney of G3S^Tg/+^*Gla*^−/y^ Mice After 3.5 Months of AAV-GLA Treatment; Table S1: Summary of mouse age, genotype, and group sizes for all experimental conditions.
