# Supplementary material for: Comparative Evaluation of AAV8 and AAV9 Gene Therapy in Fabry Knockout (Gla−/y) and Symptomatic (G3STg/+Gla−/y) Murine Models
Source: Genes (Basel). 2025 Jun 29;16(7):766. doi: 10.3390/genes16070766 (PMC12294720; doi:10.3390/genes16070766)
Supplement: Supplementary file 1 [file genes-16-00766-s001.zip › Supplementary Materials_v4.pdf]

Supplementary Materials:

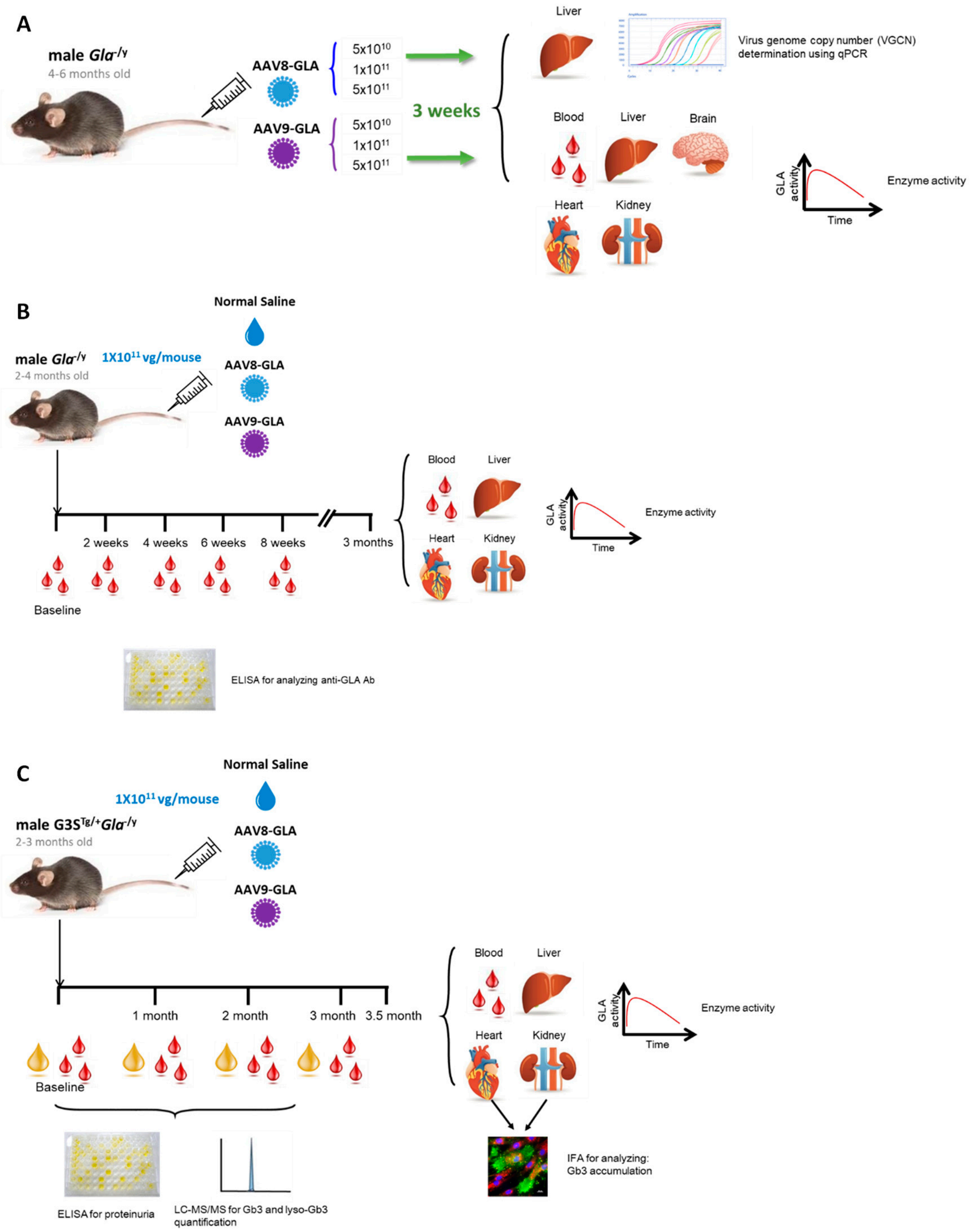

Supplementary Figure S1. Experimental design overview.

(A) Dose-Finding Study for Optimal AAV-GLA Administration. (B) Study Timeline for Anti-GLA Antibody Monitoring and Endpoint Enzyme Activity Measurement. (C) Time Course of Disease Biomarkers and Terminal GLA enzyme activity and Gb3 Accumulation Analysis

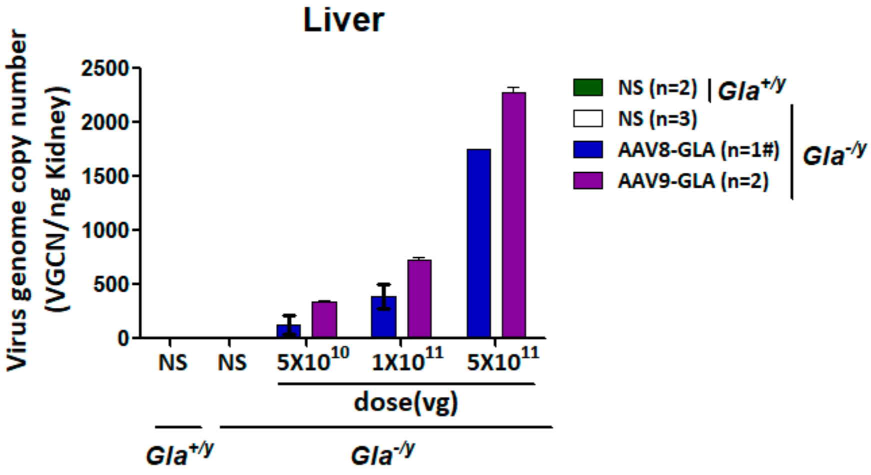

Supplementary Figure S2. Viral vector bio-distribution in tissues of *Gla*$^{-/y}$ mice following AAV-GLA administration.

Gla$^{-/y}$ male mice (4–6 months old) received AAV8-GLA or AAV9-GLA at doses of 5×10^{10}, 1×10^{11}, or 5×10^{11} vg/mouse. Livers were collected three weeks post-injection for viral genome copy number analysis. Data are presented as mean ± SD. #: In this group, two out of three treated mice died shortly after injection; therefore, only one data point was available, and standard deviation (SD) is not shown.

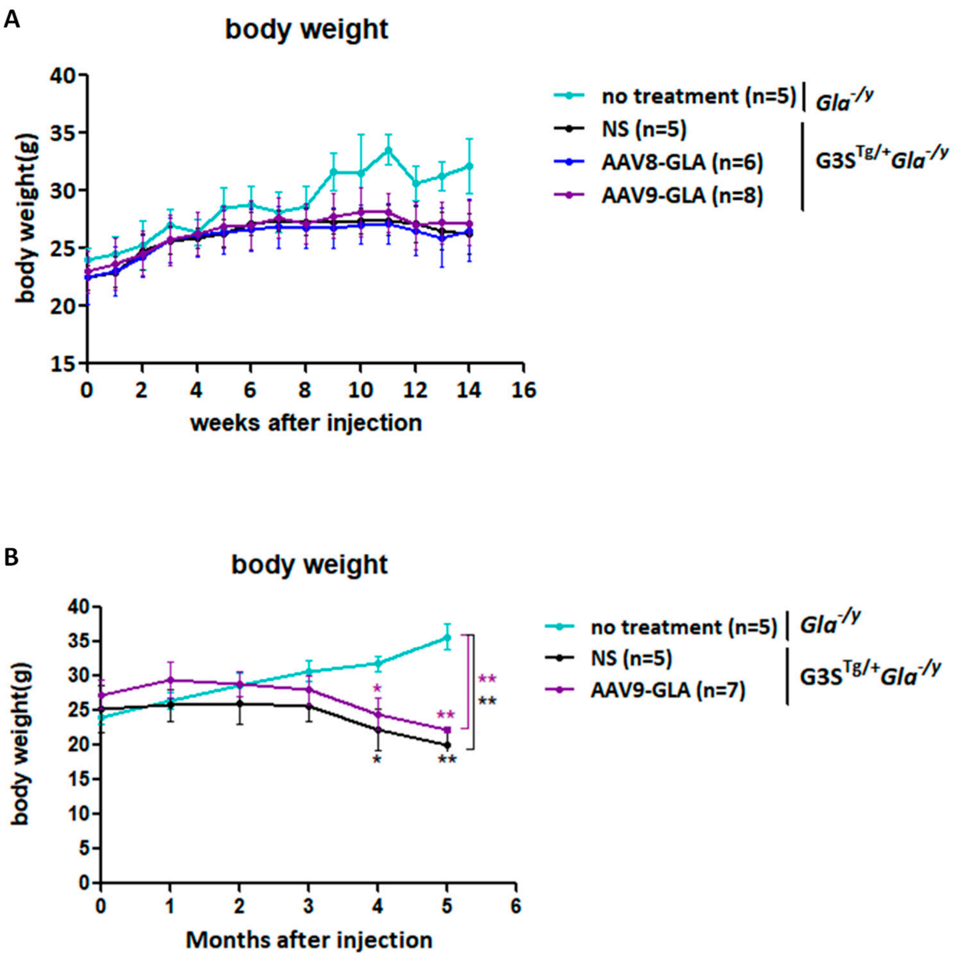

Supplementary Figure S3. Body weight monitoring of G3S$^{Tg/+}$*Gla*$^{-/y}$ mice during 3.5 months (A) and 5 months (B) of AAV-GLA treatment.

G3S$^{Tg/+}$*Gla*$^{-/y}$ male mice (2–3 months old) received a single intravenous injection of AAV8-GLA or AAV9-GLA at $1×10^{11}$ vg/mouse. Body weight was monitored weekly for 3.5 months and monthly for 5 months post-treatment. Data are presented as mean ± SD. $p < 0.05$ (*), $p < 0.01$ (**), $p < 0.001$ (***).

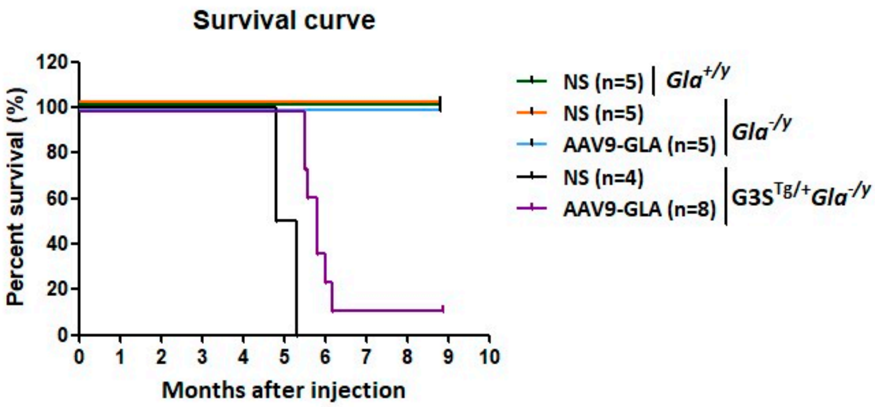

Supplementary Figure S4. Survival curve of mice monitored over 9 months of AAV9-GLA treatment.

Gla$^{-/y}$ and G3S$^{Tg/+}$*Gla*$^{-/y}$ male mice (2–3 months old) received a single intravenous injection of AAV9-GLA at 1×10^{11} vg/mouse. Mouse survival was monitored daily for a total duration of 9 months.

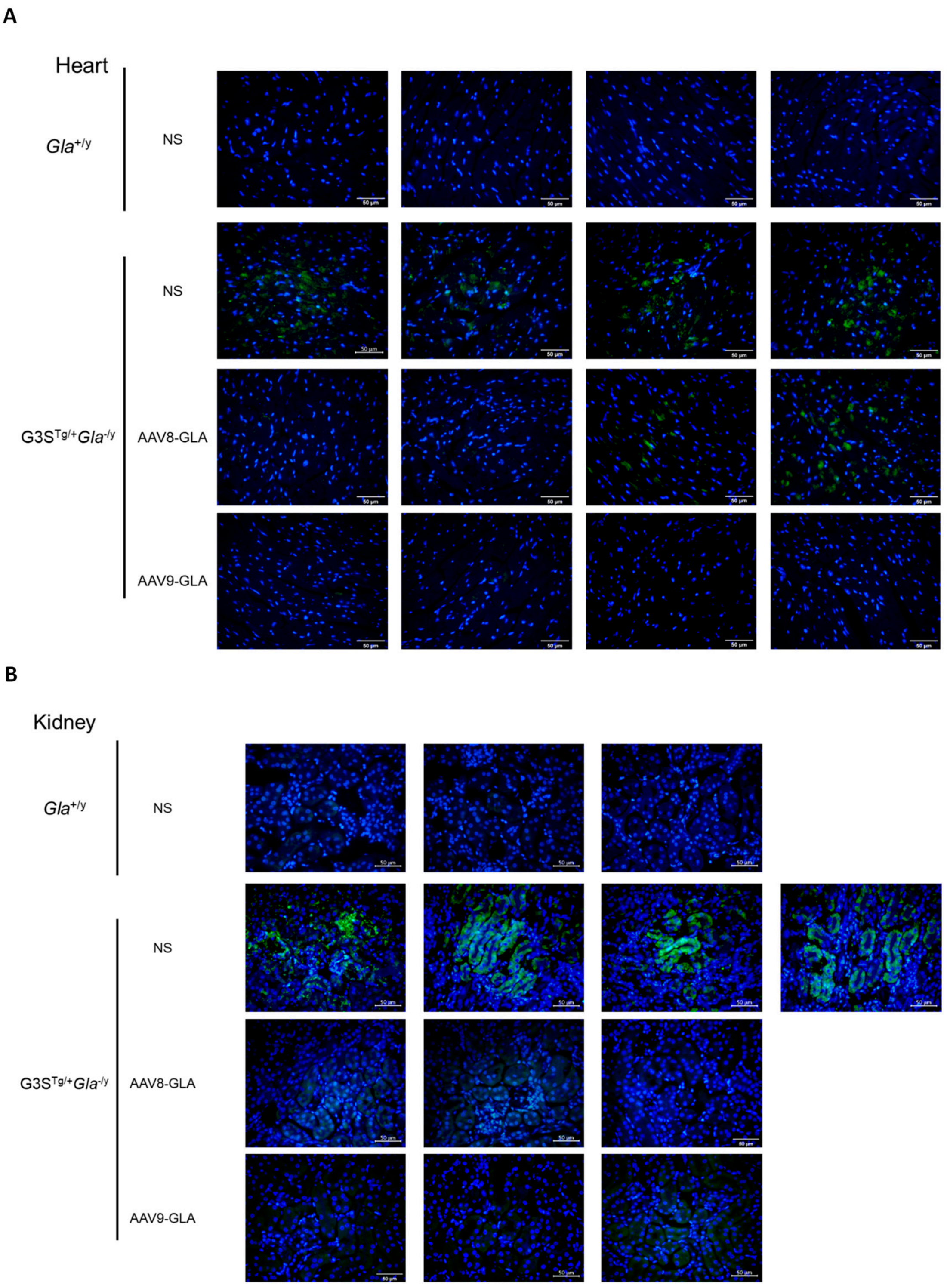

Supplementary Figure S5. Additional figure demonstrating Gb3 Clearance in Heart and Kidney of G3S$^{Tg/+}$*Gla*$^{-/y}$ Mice After 3.5 Months of AAV-GLA Treatment
