# Supplementary material for: Comparative Evaluation of AAV8 and AAV9 Gene Therapy in Fabry Knockout (Gla−/y) and Symptomatic (G3STg/+Gla−/y) Murine Models
Source: Genes (Basel). 2025 Jun 29;16(7):766. doi: 10.3390/genes16070766 (PMC12294720; doi:10.3390/genes16070766)
Supplement: Supplementary file 1 [file genes-16-00766-s001.zip › Supplementary Table.pdf]

	Figure 1 / Figure S2							Figure 2				Figure 7			
Age(months)	4-6 months							2-4 months				2-4 months			
Treatment groups / Genotype	NS	NS	AAV8			AAV8		NS	NS	AAV8	AAV9	NS	NS	AAV8	AAV9
			$5x10^{10}$	$1x10^{11}$	$5x10^{11}$	$5x10^{10}$	$1x10^{11}$			$1x10^{11}$	$1x10^{11}$			$1x10^{11}$	$1x10^{11}$
$GLA^{+/y}$	2	-	-	-	-	-	-	5	-	-	-	6	-	-	-
$GLA^{-/y}$	-	3	3	3	1[#]	2	2	-	4	5	5	-	5	6	6

	Figure 3				Figure 4			Figure 5				Figure 6			
Age(months)	2-3 month				2-3 month			2-3 month				2-3 month			
Treatment groups / Genotype	NS	NS	AAV8	AAV9	NS	AAV8	AAV9	NS	NS	AAV8	AAV9	NA	NS	AAV8	AAV9
			$1x10^{11}$	$1x10^{11}$		$1x10^{11}$	$1x10^{11}$			$1x10^{11}$	$1x10^{11}$			$1x10^{11}$	$1x10^{11}$
$GLA^{+/y}$	5	-	-	-	-	-	-	3	-	-	-	-	-	-	-
$G3S^{Tg/+}GLA^{-/y}$	-	8	6	7	4	4	4	-	3	3	3	9	6	7	7

	Figure S3A				Figure S3B			Figure S4				
Age(months)	2-3 month				2-3 month			2-3 month				
Treatment groups / Genotype	NA	NS	AAV8	AAV9	NA	NS	AAV9	NS	NS	AAV9	NS	AAV9
			$1x10^{11}$	$1x10^{11}$			$1x10^{11}$			$1x10^{11}$		$1x10^{11}$
GLA$^{+/y}$	-	-	-	-	-	-	-	5	-	-	-	-
GLA$^{-/y}$	5	-	-	-	5	-	-	-	5	5	-	-
G3S$^{Tg/+}$*GLA*$^{-/y}$	-	5	6	8	-	5	7	-	-	-	4	8

Supplementary Table S1. Summary of mouse age, genotype, and group sizes for all experimental conditions.
Abbreviations: NA, no treatment control; NS, normal saline; AAV8, AAV8-GLA; AAV9, AAV9-GLA.
#starting with 3 mice, but 2 die shortly after AAV8 administration
